# Bioprinting extracellular vesicles as a "cell-free" regenerative medicine approach

**DOI:** 10.20517/evcna.2023.19

**Published:** 2023-05-23

**Authors:** Kexin Jiao, Chun Liu, Saraswat Basu, Nimal Raveendran, Tamaki Nakano, Sašo Ivanovski, Pingping Han

**Affiliations:** ^1^The University of Queensland, Faculty of Health and Behavioural Sciences, School of Dentistry, Center for Oral-facial Regeneration, Rehabilitation and Reconstruction (COR3), Epigenetics nanodiagnostic and therapeutic group, Brisbane 4006, QLD, Australia.; ^2^The University of Queensland, Faculty of Health and Behavioural Sciences, School of Dentistry, Brisbane 4006, QLD, Australia.; ^3^Hokkaido University, Institute for Catalysis (ICAT), N21 W10, Kita-ku, Sapporo 001-0021, Japan.

**Keywords:** 3D bioprinting, small extracellular vesicles, bioprinted sEVs, regenerative medicine

## Abstract

Regenerative medicine involves the restoration of tissue or organ function via the regeneration of these structures. As promising regenerative medicine approaches, either extracellular vesicles (EVs) or bioprinting are emerging stars to regenerate various tissues and organs (i.e., bone and cardiac tissues). Emerging as highly attractive cell-free, off-the-shelf nanotherapeutic agents for tissue regeneration, EVs are bilayered lipid membrane particles that are secreted by all living cells and play a critical role as cell-to-cell communicators through an exchange of EV cargos of protein, genetic materials, and other biological components. 3D bioprinting, combining 3D printing and biology, is a state-of-the-art additive manufacturing technology that uses computer-aided processes to enable simultaneous patterning of 3D cells and tissue constructs in bioinks. Although developing an effective system for targeted EVs delivery remains challenging, 3D bioprinting may offer a promising means to improve EVs delivery efficiency with controlled loading and release. The potential application of 3D bioprinted EVs to regenerate tissues has attracted attention over the past few years. As such, it is timely to explore the potential and associated challenges of utilizing 3D bioprinted EVs as a novel "cell-free" alternative regenerative medicine approach. In this review, we describe the biogenesis and composition of EVs, and the challenge of isolating and characterizing small EVs - sEVs (< 200 nm). Common 3D bioprinting techniques are outlined and the issue of bioink printability is explored. After applying the following search strategy in PubMed: "bioprinted exosomes" or "3D bioprinted extracellular vesicles", eight studies utilizing bioprinted EVs were found that have been included in this scoping review. Current studies utilizing bioprinted sEVs for various *in vitro* and *in vivo* tissue regeneration applications, including angiogenesis, osteogenesis, immunomodulation, chondrogenesis and myogenesis, are discussed. Finally, we explore the current challenges and provide an outlook on possible refinements for bioprinted sEVs applications.

## INTRODUCTION

Regenerative medicine aims to cure diseases and guide the reconstruction of malformations and traumatic injuries. Regenerative medicine approaches include transplantation of stem cells or biological molecules *in vivo*, replacement of organs or tissues in whole or in part with cellular structures grown *ex vivo*, and using bioactive biomaterials to harness innate regenerative processes for restoration of organ or tissue function^[1]^. The "cell-free" approach harnesses the therapeutic potential of bioactive molecules (i.e., extracellular vesicles, growth factor) without involving live cells. It utilizes techniques like injectable hydrogels, scaffolds, and bioprinting to deliver these molecules for targeted release, aiding damaged or diseased tissues^[2]^. As a new class of regenerative approach, extracellular vesicles and 3D bioprinting are the focus of this review.

Extracellular vesicles (EVs) are an emerging means of cell-to-cell communication that is important for a wide range of biological and therapeutic applications. According to the International Society for Extracellular Vesicles (ISEV), the term "EV" is a general nomenclature for cell-secreted membrane-bound bilayered lipid membrane vesicles that contain molecules secreted from living cells into the extracellular space^[3,4]^. These molecular components include proteins, nucleic acids and lipids, which enable intercellular communication of a parent cell’s biological information to a recipient cell^[4,5]^. Both eukaryotic and prokaryotic (i.e., bacteria) cells can release EVs with varying biological materials that modulate signaling pathways in the recipient cell. Increasing evidence suggests that EVs are involved with physiological and pathological developments such as tumor metastasis, tissue homoeostasis, and inflammatory diseases^[5-9]^. However, therapeutic delivery of stable EVs into target sites (tissues and cells) and controlled release via appropriate carriers (e.g., hydrogel) remains elusive. This review focuses on an emerging state-of-the-art technique - three-dimensional (3D) bioprinting - as an effective EVs delivery system for therapeutic applications.

3D bioprinting, or extended additive manufacturing (AM), utilizes computer-aided processes to enable automated simultaneous layer-by-layer precise patterning of biomaterials, biochemicals, live cells and growth factors to achieve a controlled functional construct or structure^[10-12]^. 3D bioprinting utilizes computer-aided design/computer-aided manufacturing (CAD/CAM) to fabricate sophisticated 3D biocompatible structures by automating the deposition of biological material or cells within a substrate^[13-15]^. The printed 3D structures, comprising living cells, biomaterials, and biological molecules, are fabricated in a bioink that retains the bioactivity of the structure after printing^[11,12]^. 3D bioprinting is emerging as a novel regenerative medicine approach to meet the specific requirements for bioengineered tissues and organs^[10]^, by fabricating tissue-engineered scaffolds with tunable geometry, size, porosity, and interconnectivity^[12,16]^ that effectively facilitate the regeneration of new, or the repair of damaged, tissues^[10,16]^. Three main technologies have been used for bioprinting: inkjet^[17]^, pressure-assisted microextrusion^[18]^, and laser-assisted bioprinting^[11]^. There are advantages and disadvantages with each bioprinting technique, with limitations associated with bioink design requirements and strategies, which will be explored in the following section. Bioprinted live cells have been widely used for regenerating various tissues^[19]^, such as bone^[20]^, cartilage^[21]^, blood vessels^[22]^, and periodontal structures^[23]^. However, obtaining sufficient cell numbers *in vitro* and retaining cell function after printing remains challenging. It is of great significance to develop alternative "cell-free" biological molecules (i.e., EVs) for bioprinting.

There are limited studies that utilize bioprinted EVs as an alternative "cell-free" regenerative medicine approach for various tissue regeneration applications. A recent perspective review provided insights into the future potential of bioprinted EVs for clinical application^[24]^, but did not describe 3D bioprinting and current studies. Herein, current pre-clinical *in vivo* studies reporting the potential application of bioprinted EVs in tissue engineering and regeneration are reviewed. The following sections will present an overview of the biogenesis, isolation, and characterization of EVs (small EVs, also named exosomes), then discuss current advances in bioprinting strategies, the potential applications of 3D bioprinted EVs as novel therapeutics, the limitations and gaps of current knowledge, and future research directions.

## BIOGENESIS, COMPONENTS, AND ISOLATION OF CELL-DERIVED EVS

As stated by the *Minimal information for studies of extracellular vesicles 2018* (MISEV2018), EVs have been defined based on physical features of size, density, biochemical composition, or cell of origin, and can be classified into three subtypes: apoptotic bodies, microvesicles, and exosomes^[3,4]^. Despite the nomenclature of EVs still evolving, the ISEV suggests naming EVs based on size - small EVs (< 200 nm) and medium/large EVs (> 200 nm) - unless researchers can demonstrate the endosomal or ectosomal origins of their EVs secretion^[5]^. Moreover, it is challenging to define EVs based only on EVs size since three EVs subtypes overlap in this parameter. For example, small extracellular vesicles (sEVs, also known as exosomes) are smaller than 200 nm, microvesicles (MVs) are in the range of 50-1,000 nm and apoptotic bodies (ApoBD) with diameters of 50-2,000 nm^[5]^. Standardization of nomenclature for each EV subtype remains a challenge.

### Biogenesis of EVs

**Figure 1:**
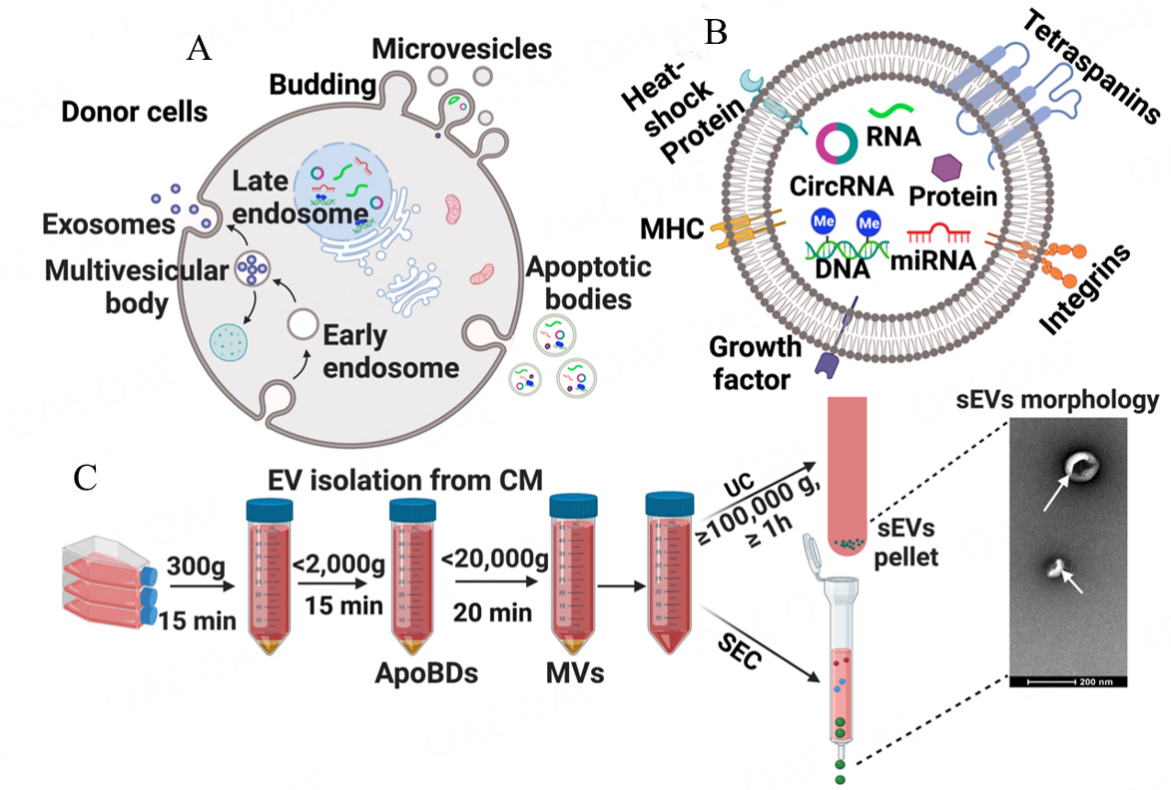
EVs Biogenesis, components, and cell-derived EVs solation method. (A) Biogenesis of Extracellular vehicles (EVs); (B) components of EVs; (C) Common EVs isolation steps using a serial centrifuge and sEVs isolation by either UC or SEC method prior to TEM analysis of sEVs morphology. MHC: Major histocompatibility complex; CM: condition media; ApoBDs: Apoptotic bodies; MVs: Multivesicular body; SEC: Size Exclusion Chromatography; UC: ultracentrifugation.

As the largest EVs, ApoBD is formed by the outward blebbing of an apoptotic cell membrane, resulting in phosphatidylserine-rich vesicles [Figure 1A]^[25]^. MVs are phosphatidylserine and cholesterol-rich particles that are shed from the plasma membrane. In this review, we will define "EV" as a generic term for all EVs, while we identify "sEVs" for exosomes or EVs that are smaller than 200 nm.

Most current studies investigate sEVs that are generated via the endocytic pathway^[4]^. Indeed, EVs can be uptaken by membrane fusion, endocytosis or receptors on the cell surface^[5,26]^. Exosomes are generated *via* inward budding of endomembrane structures such as early endosomes, resulting in intraluminal vesicles (ILVs) in multivesicular bodies (MVBs)^[27]^. Early endosomes mature in several ways forming late endosomes, a complex process subject to ongoing exploration. Late endosomes are the final step to release exosomes through the fusion of MVBs with the plasma membrane that coordinates the extracellular release of sEVs [Figure 1A]^[4,28,29]^.

### EVs composition and isolation methods

Importantly, EVs carry a cargo of various biological molecules from their patent cells, including tetraspanins (i.e., CD9, CD81, and CD63), lipids, integrins, major histocompatibility complex (MHC), heat-shock protein (HSP), growth factors, circular RNAs, circular RNA (circRNA), microRNAs (miRNA), mRNA, proteins, and long non-coding RNAs, as well as genomic DNA [Figure 1B]^[7,27,30-32]^. These EVs compositions make them the best candidate for both diagnostic and therapeutic tools.

Proper isolation and characterization of EVs are essential for EV therapy^[33]^. Isolation techniques ensure purity, eliminating contaminants that can impact therapeutic efficacy. Characterization reveals EV composition and functional properties, aiding in targeted delivery. Adhering to guidelines promotes standardization and comparability. Overall, these steps maximize the therapeutic potential and facilitate the successful translation of EV-based therapies to the clinic.

Currently, there are no standard methods for EVs isolation; it is well-accepted that apoptotic bodies and microvesicles can be obtained via serial centrifugation [Figure 1C] . In general, the current gold-standard method for isolation of sEVs is ultracentrifugation; other techniques such as ultrafiltration, precipitating agents (i.e., polythene glycol), immunoaffinity capture, microfluidics, and size-exclusion chromatography (SEC) have emerged as viable options^[34,35]^. As discussed in our previous review^[36]^, it is critical to consider several factors for downstream EVs isolation *in vitro* from conditioned media (CM), such as primary cell source (donor gender/age/health status), passage number, CM volume/change frequency and composition (EVs-depleted FBS or FBS-free), and CM harvesting conditions. In general, cell-derived EVs isolation protocol is similar to that for oral fluid^[37]^. First, CM is collected from cell culture and centrifuged for 15 mins at 300 *g* to remove cell debris before being centrifuged at < 2,000 *g* for 15 mins to pellet apoptotic bodies. Then, microvesicles can be obtained from a centrifuge at < 20,000g for 20 mins. Last, sEVs will be enriched by either ultracentrifuge (UC, at >100,000 *g* for > 1 h) or a size exclusion chromatography column. Then, purified sEVs can be characterized with cup-shaped morphology by transmission electron microscopy (TEM). It is noted that sEVs can also be isolated by precipitation-based isolation (i.e., ExoQuick), immunoaffinity chromatography, and ultrafiltration.

As suggested by MISEV2018 guidelines^[4]^, three aspects of EVs characterization need to be performed for all EV studies: cup-shaped morphology, EVs-enriched protein analysis, and EVs size distribution. Thus, TEM (for morphology), nanoparticle tracking analysis (NTA, for size distribution), dynamic light scattering (DLS, for size distribution), enzyme-linked immunosorbent assay (ELISA), for EV-protein), western blot (WB, for EV-protein) and nanoscale flow cytometry (for EV-protein) be used to characterize EVs after isolation^[4,35,38]^.

### Circulating sEVs as therapeutic tools

EVs are emerging as powerful biomarkers for disease diagnosis and prognosis, such as periodontal disease^[37,39-43]^ and cancer^[44,45]^. sEVs are circulating and enriched in different biofluids, including saliva^[46,47]^, urine, bronchial fluid, cerebral spinal fluid, breast milk^[48]^, serum^[49]^, amniotic fluid, and plasma^[50]^, and are easy to collect as liquid biopsies for disease diagnosis^[37,50,51]^ via several different methods[52]. Non-invasive biofluids-saliva and gingival crevicular fluids^[53]^ are potential liquid biopsies to study EVs and other relevant biological molecules^[53-55]^. Current research strongly supports the potential of EVs as a regenerative medicine intervention due to their biological components, or as a therapeutic delivery vehicle to treat diseases^[8,56]^. The therapeutic potential of EVs has been emphasized in multiple reviews, showcasing their efficacy in addressing various diseases, including skeletal diseases, cardiac diseases, brain diseases, and cancers^[33,57,58]^. These findings highlight the promising role of EVs as a versatile therapeutic modality across diverse pathological conditions. A new study identifies EVs as a next-generation drug delivery platform after comparing EVs with well-known liposomes and discusses the development of EV-based drug delivery systems^[59]^. Tissue engineering technologies, such as hydrogels, nanotubes, or polymeric biomaterials, have been used to stabilize EVs and efficient EVs delivery^[60,61]^. Advances in EVs delivery systems, such as the application of 3D bioprinting, the focus of this review, are emerging. Bioprinted EVs have attracted considerable interest in tissue regeneration over the past few years, and with no standard methods for bioprinting EVs, recent studies of bioprinted EVs and their potential regenerative applications are summarized in this review.

## THE GENERAL CONCEPT OF 3D BIOPRINTING

3D bioprinting is an AM technique that involves controlled deposition of bioinks containing cells, growth factors, and other bioactive molecules to create 3D tissue-like constructs that mimic in vivo tissue properties^[14,62]^. An ideal bioink should be assessed for printability, mechanical properties, and biocompatibility. Bioprinted layer-by-layer constructs consist of interconnected pores that are optimal for the infusion of gas and nutrients and, importantly, cellular communication^[15,63]^. 3D Bioprinting can be customized to produce the desired shape, size, internal porosity, and interconnectivity for the fabrication of scaffolds for various tissue-engineering applications^[64]^. An ideal bioprinting strategy should: (A) support viability and architecture of encapsulated cells or biomolecules; (B) be compatible with diverse bioinks with different viscosities and crosslinking groups; (C) enable concise control of spatial pattern arrangements of scaffolding materials, biological factors and cell over clinically relevant dimensions. Herein, we describe common bioinks and printability and various bioprinting strategies.

### Bioinks and printability

**Figure 2:**
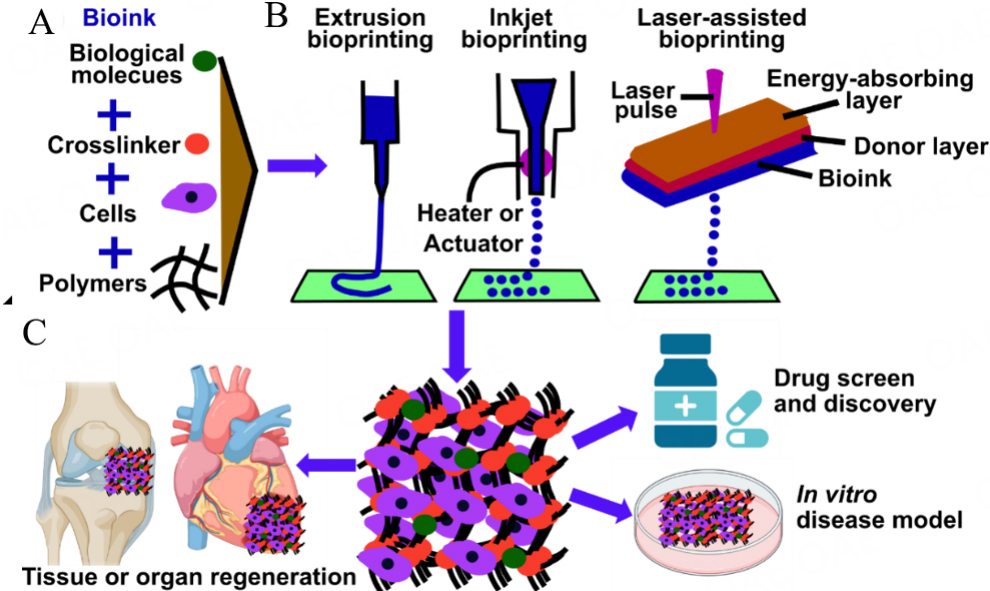
Schematics of bioinks (A), 3D bioprinting methods (B) and the application of 3D bioprinting (C). (A) Bioink typically includes biological molecules, live cells, cross-linker, and polymers (GelMA hydrogel). (B) Current widely used 3D bioprinting techniques of extrusion, inkjet and laser-assisted bioprinting. (C) Three main applications of 3D bioprinting lie in drug screening, *in vitro* disease models and tissue regeneration.

Requiring properties to meet specific conditions and requirements, one of the greatest and most significant challenges is the utilization of an appropriate bioink. Bioinks consist of biomaterials, live cells and biomolecules, which are a key component of 3D bioprinting [Figure 2A] . One of the key challenges in the 3D bioprinting field is to find suitable materials that are not only biocompatible but also provide the desired mechanical and functional properties for targeted tissue constructs. Cell-loaded bioinks are hydrogel-based, as hydrogels have a high-water content required for cell viability and protection of the cells from manufacturing-generated stresses^[11]^. The primary material properties of a bioink that need to be evaluated before printing include its viscosity, gelation, rheological properties, and crosslinking capabilities^[11,12,64]^. Bioinks can be natural materials, such as collagen, fibrin, hyaluronic acid (HA)-, agarose-, silk-, glycerol-, cellulose-, and alginate-based bioinks^[65]^, that might be utilized in the form of hydrogels, or synthetic materials such as PCL, polylactide (PLA), polyglycolide (PGA) and polyethylene glycol (PEG) polymers^[66]^. A growing body of research shows that the use of glycerol, as an often-used material, may be rationalized by the fact that it is non-toxic and generally elicits limited immune responses. Also, glycerol may have advantages, including adjustable viscosity as an aqueous solution that can be tuned by changing concentration as well as chemical stability, which would be suited for bioprinting applications. From the perspective of materials science, poly(ethylene glycol) [IUPAC name: poly(oxyethane-1,2-diyl)] (PEG) derivatives may also be a candidate component of bioinks. It is often biologically inert and has been used as a modifier of peptides, proteins, and oligonucleotides through a chemical bond formation called “PEGylation”^[67]^, since the first example of such application was reported in 1977^[68]^. A range of PEG derivatives is currently available from commercial sources that may allow a much wider scope of physicochemical properties compared with glycerol, a rationale for continuing to explore the use of PEG derivatives in the bioprinting field.

Recent advancements in bioprinting have expanded the materials used in bioinks beyond cell-laden hydrogels, such as incorporating bioactive molecules to enhance cell behavior and tissue regeneration. Growth factors such as transforming growth factor-beta (TGF-β), bone morphogenetic protein (BMP) and vascular endothelial growth factor (VEGF) are included to promote cell proliferation, differentiation, and tissue-specific functionality^[69,70]^. Bioinks also incorporate ECM proteins or protein peptides like arginine-glycine-aspartic acid (RGD), mimicking the natural tissue environment to promote cell adhesion, migration, and tissue organization^[71]^. These multifunctional bioinks represent a significant breakthrough, enabling the fabrication of complex, functional tissues, and organs.

Printability is the most important parameter when determining material qualities. Printability is dependent on two aspects^[72,73]^: (i) the viscoelastic properties of the bioink formulation and (ii) the shape fidelity associated with the mechanical strength of the printed construct to self-sustain a 3D structure post-printing. Printability may be affected by bioink viscosity, gelation, or crosslinking capabilities, depending on the printing method^[12]^. There is a significant need for creating suitable cell-laden bioink materials with “printability” for 3D bioprinting, which is associated with required physicomechanical properties and cellular compatibility^[20,74,75]^.

### Different techniques of bioprinting

Inkjet-based bioprinting, extrusion-based bioprinting, and laser-based bioprinting are common 3D bioprinting technologies^[12,76]^ [Figure 2B]. These widely used 3D bioprinting techniques have been utilized for regenerative tissues and organs *in vitro* and *in vivo*^[77]^ and emerging as a promising and innovative fabrication strategy to precisely position biologics in the prescribed 3D hierarchal organization to create artificial multicellular tissues/organs.

Laser-based bioprinting is a process of curing (polymerizing) a polymer-filled vat using a high-intensity laser such as ultraviolet, monochromatic laser, or visible light. When exposed to light energy, photoinitiators generate reactive agents that react with monomers in a substance to synthesize polymer chains. Different wavelength ranges activate photoinitiators; some are triggered by UV, while others are triggered by visible light^[11]^. The polymerization that occurs when the energy beam reaches the liquid boundary induces a phase transition into a solid. The solid layer lowers, while a liquid polymer layer comes to the top^[11]^. The new layer is then exposed to an energy beam that solidifies it^[64,78,79]^. The liquid vat is placed on a moveable stage that is programmed to move on the Z-axis according to a CAD, resulting in a layer-by-layer structure^[76]^. SLA provides great precision and resolution but is limited by the lack of biocompatible resin options^[76,78]^. Using the SLA technique, human umbilical vein endothelial cells (HUVECs)^[80]^, NIH-3T3 fibroblasts cell line^[81]^, and 344SQ lung adenocarcinoma cells^[82]^ have been bioprinted for various applications.

Inkjet printing injects liquid droplets of a target substance with a controlled bioink volume^[83]^. This technique deposits polymeric solutions, colloidal suspensions, and cell suspensions with low viscosities at high shear rates in the form of droplets^[12]^. A cartridge is generally included with inkjet equipment and might incorporate several dispensing principles (piezoelectric element, thermal film resistor, electromagnetic pin actuator, or acoustic ejector)^[83,84]^. By applying liquid phase materials, inkjet printing is a material-conserving deposition process in which bioinks are composed of a solute that has been dissolved or otherwise dispersed in a solvent^[83]^. The variety of bioinks available for this printing is limited, and they must have a low viscosity.

Extrusion bioprinting is one of the most frequently used and inexpensive biological and non-biological 3D bioprinting methods^[10]^. Typical components of a microextrusion bioprinter comprise a temperature-controlled material-handling and dispensing system and stage, with one or both capable of movement along the x, y, and z axes, a fiberoptic light source to illuminate the deposition area and/or for photoinitiator activation, a video camera for x-y-z command and control, and a piezoelectric humidifier^[10]^. Microextrusion printers operate by extruding and dispensing the material onto a substrate by a microextrusion head. Microextrusion printers work by extruding a material that is then deposited onto a substrate by a robotically controlled microextrusion head. Instead of liquid droplets, microextrusion generates continuous beads of material. Hydrogels, biocompatible copolymers, and cell spheroids are all acceptable with microextrusion printers^[84]^. The capacity to deposit very high cell densities is the real advantage of the microextrusion bioprinting technique. Mechanical microextrusion is the most prevalent approach for scaffold-less tissue spheroid bioprinting^[10,62]^. However, the printing resolution (200-2,000 μm) is one of the main limitations of this technique, which requires optimization of processing conditions (e.g., flow rate and deposition velocity) and bioink properties (e.g. rheological properties-wettability, surface tension, and cell density).

The advantages and disadvantages of standard methods are summarized in Table 1. As such, depending on the complexity of the final tissue construct, different 3D printing methods and bioink can be selected.

**Table 1:** Advantages and disadvantages of 3D bioprinting techniques

**Printing method**	**Advantages**	**Disadvantages**
Extrusion bioprinting	Printing speed and structures can be highly controlled Mutil-material/cell printing is allowed using multiple printing heads Ability to deposit high cell densities	Shear stress can impact cell viability Limited printing resolution (200-2,000 µm) Nozzle clogging Slow printing process
Inkjet-based bioprinting	Fast printing speed Potential to print different concentration gradients of cells High precision Low cost Gentle to printed cells	Requires low-viscosity materials Frequent nozzle clogging Unreliable cell encapsulation Limitation of cell density Low resolution
Laser-based bioprinting	Can be used with viscous materials Highly resolution (80-140 µm) Nozzle-free technique, so not prone to clogging problem High cell viability	Only one cell type can be printed at a time UV damage to cells A small range of bioinks Low overall flow rate Slow process

### The therapeutic application of 3D bioprinting

3D bioprinting has been applied in various applications, such as tissue engineering, *in vitro* disease models, and drug screening [Figure 2C] .

Various tissue constructs to mimic native tissue and organs - bone, vascular, skin, cartilage, and neural structures have been successfully manufactured using several 3D bioprinting approaches (reviewed in^[62,77,85-87]^). For example, bioengineered cardiac tissue *via* 3D bioprinting technology is gaining increasing importance owing to the rising numbers of heart attacks, heart failure, toxicology research, drug testing and screening, and personalized medicine^[85,88-90]^. Noor *et al.* successfully printed cellularized human hearts with a natural architecture, utilizing an extrusion-based bioprinting technique and personalized bioink (patient’s decellularized omentum tissues and cardiomyocytes)^[91]^. Inkjet and extrusion-based bioprinting were used to print mesenchymal stem cells in nanocellulose or GelMA to promote osteogenesis and bone tissue regeneration^[88,92]^. Recent reviews have extensively documented the various applications of 3D bioprinting in tissue regeneration, particularly in the fields of skin, muscle, cardiac, and orthopedic tissue regeneration^[77,93-96]^. These comprehensive studies have provided valuable insights into the advancements and potential of 3D bioprinting techniques in facilitating the regeneration of these specific tissues. By exploring the innovative strategies and biomaterials employed in bioprinting, as well as the integration of biologically relevant cells, growth factors and other biomolecules, these reviews have shed light on the promising therapeutic outcomes and prospects for these tissue types. Additional studies are needed to provide all required features for each target tissue.

### 3D bioprinting for in vitro disease model and drug screening

To understand disease progression and normal functioning of the body, it is imperative to reconstruct the *in-vivo* environment in an *in vitro* setting. Here, the use of 3D bioprinting becomes vital. This technology provides us with the ability to recreate a three-dimensional model, one which is seen inside a body.

In recent years there have been a significant number of studies that have incorporated 3D bioprinting to create a three-dimensional culture system and tested its efficacy and viability in recapitulating *in vivo* conditions. One such study used a combination of novel peptide-modified biopolymer, gellan gum-RGD (RGD-GG) and primary cortical neurons as their bioink and 3D bioprinted constructs that would mimic a brain-like structure. The authors confirmed that the modified biopolymer facilitated primary cell proliferation, and the high cell viability of the model means that this study can be used to study a range of neurodegenerative diseases, cell behavior studies, and brain injuries^[97]^.

Bioprinting also has the potential to be used in drug screening, as demonstrated by Mondal *et al.* Their 3D bioprinted spheroid model with non-small cell lung cancer (NSCLC) cell, PDX and lung cancer-associated fibroblasts, constructed on a sodium alginate/gelatin scaffold *via* extrusion-based 3D bioprinting could be used as a model for high throughput drug testing^[98]^. A study by the same group also bioprinted cancer spheroid with high viability and proliferation^[99]^. Bioprinting can not only help us to understand disease progression or *in vivo* mechanics, but it can also help us to treat certain aberrations in the body. As seen in one study, the researchers created a 3D bioprinted patch containing human cardiac-derived progenitor cells in a hyaluronic acid/gelatin-based matrix. In a lab setting, the cells inside showed high viability and retained their proliferative properties and differentiation capability. When this patch was transplanted in a myocardial infraction mouse model, preservation of cardiac function and significant reduction in adverse modeling were observed^[100]^. The application of bioprinting has also been studied for tissue regeneration, and quite extensively for skin regeneration. The study done by Stefanie’s group bioprinted a skin construct using laser-assisted bioprinting. The construct had fibroblasts and keratinocytes over a Matriderm layer. The construct was placed in a mouse model and showed tissue regeneration, and blood vessels were also seen to be growing towards the direction of the construct from the skin wound^[101]^. This study paves the way for more research that can incorporate a wide variety of cells for a more complex model and better.

Although bioprinting possesses significant promise for regenerative medicine, living cells as part of bioink formulation pose significant challenges for clinical translation, such as the regulatory and cost implications of cell culture and storage *in vitro*, and the issues of nutrient access, immune rejection, and engraftment upon implantation *in vivo*. “Cell-free” molecules, such as growth factors, blood products, bioceramics, nanoparticles and EVs, can be bioprinted to overcome these challenges. This scoping review will explore the potential of bioprinted EVs as a "cell-free" regenerative medicine approach.

## BIOPRINTED EVS AS A "CELL-FREE" REGENERATIVE MEDICINE APPROACH

While tissue regeneration applications for 3D bioprinted sEVs have emerged over the past several years, only a limited number of studies have been conducted. It is known that cell source, EVs enrichment and characterization are critical for downstream applications. Here, we summarized cell source, conditional media collection parameters, and current methods of EVs isolation and characterization, as well as a detailed application for each selected study.

### Study search strategy

English titles and abstracts from all retrieved records were screened for potentially eligible studies by one reviewer (K.J.), with the following keywords and other free terms: "Bioprinted exosomes" or "3D bioprinted extracellular vesicles". The search was conducted using three electronic databases: Google Scholar, PubMed, and EMBASE. Filter for the 2019-2023 date was placed to ensure that all current relevant articles were included.

### Cell source of bioprinted EVs

The current review includes a total of seven studies in the field that investigated sEVs secreted from primary mesenchymal stem cells (MSCs), human adipose tissue-derived mesenchymal stem cells (hADSCs), or cell lines such as human umbilical vein endothelial cells (HUVEC), head and neck squamous cell carcinomas (HNSCCs), human leukemia monocytic cell line - THP1, and murine monocytic cell line - J774A.1 *in vitro* [Table 2]. It is noted that bioprinted EV-associated protein array was utilized for cancer diagnosis in a study^[102]^, which is not the therapeutic application of bioprinted EVs.

**Table 2:** Representative studies of bioprinted sEVs for various regenerative medicine applications

**Reference**	**EV isolation and characterization**	**Bioprinted EV protocols**	**Key findings**
Kang *et al.*^[109]^	**Cell source:** human adipose-derived mesenchymal stem cells (ADSCs) **CM condition:** serum-free DMEM at 37 °C for 48 h **sEVs isolation (ultracentrifuge):** 300 × *g* (10 min), 2,000 × *g* (20 min), 10,000 × *g* (30 min), followed by serial filtrations with 0.45 and 0.22 μm filters. 100,000 × *g* for 70 min, then 100,000 × *g* for 70 min **EV characterization:** BCA, TEM, NTA	**Type of bioprinting:** pneumatic-driven microextrusion 3D bioprinter (Bio-Architect pro) **Amount of EVs in bioink:** 10 µg/mL, 20 µg/mL, and 30 µg/mL **Bioink material:** decellularized extracellular matrix (dECM), gelatin (Gel), quaterinized chitosan (QCS), and nano-hydroxyapatite (nHAp) **Bioprinting parameters:** printing speed - 8-10 mm s^-1^; Squeeze pressure: 0.24-0.27 MPa; scaffold size: 10 × 10 × 2 mm **EV release experiment:** BCA for 2 h, 12 h, 24 h, 3 d, and 7 d	**hADSCs-sEVs size:** 40-150 nm; 84.33% of EVs were released from the bioprinted EV scaffolds after 1 week ***In vitro*:** Bioprinted hADSCs-sEVs scaffold promoted osteogenesis in hBMSCs and angiogenesis in HUVECs. ***In vivo:*** Bioprinted hADSCs-sEVs enhanced bone formation and vascularization in rat skull defect after 10 weeks. Blood vessel formation was increased in bioprinted hADSCs-sEVs scaffolds at 1 week post subcutaneous implantation in mice
Born *et al*.^[104]^	**Cell source**: bone marrow-derived mesenchymal stem/stromal cells (MSCs) **CM condition**: BDMSC media with 10% EV-depleted FBS for 16 h where EV-depleted FBS was obtained by heat-inactivated (HI) at 56°C for 30 min, then HI-FBS was centrifuged at 100,000 × *g* for at least 16 h, and the supernatant was filtered through a 0.20 μm bottle top filter **sEVs isolation (ultracentrifuge)**: 1,000 × *g* for 10 min; 2000 × *g*; 10,000 × *g* for 30 min; 100,000 × *g* for 2 h **EV characterization**: TEM, WB (CD63, TSG101, and Alix)	**Type of printing**: microextrusion bioprinter **Amount of EVs in Bioink**: 8.84 µg EVs/ µL **Bioink material**: gelatin methacrylate (GelMA) **Bioprinting parameters**: diameter 6 mm and 2 mm thickness; pressure: 30 psi; speed: 2 mm/s; UV Curing: print a layer, cure a layer; cure speed: 20 mm/s; light irradiance: 850 mW/cm^2^ **EV release experiment**: For the release of the EV-loaded GelMA with 0.1% LAP/0.2% LAP, PBS was collected and replaced with 1.7 mL of fresh PBS on hours 1, 4, and 8 as well as days 1, 2, 3, 4, 7, 14, and 21; using an Exo ELISA-ULTRA Complete Kit (CD63 Detection; System Biosciences, Mountain View, CA)	sEV size: ranging from 30 to 250 nm (mean = 130 ± 51 nm) sEVs from mesenchymal dry/stromal cells (MSCs) can be incorporated into 3D-printed gelatin methacrylate (GelMA) hydrogel bio-inks and can be reduced by increasing the concentration of crosslinking agents during gelation to reduce the initial burst release of EVs ***In vitro***: Bioprinted MSC-sEVs constructs led to increased endothelial gap closure assay in HUVECs cells at 1-day post-incubation
Maiullari *et al*. ^[103]^	**Cell source**: Human umbilical vein endothelial cell (HUVEC) cell line. **CM condition**: normoxia, hypoxia, serum-free normoxia, and serum-free hypoxia (SM hypoxia) in EV-depleted FBS for 48 h. **sEVs isolation (ultracentrifuge)**: 500 *g* 15 min, 1,000 *g* 25 min, 125,000 *g* 90 mins at 4 °C **EV characterization**: NTA, WB (CD9, CD81), FACS (CD9, CD81 and CD63), ELISA (VEGF, PIGF, VEGFR1, VEGFR2) and TEM	**Type of printing**: microextrusion bioprinter (CeciliaOpenOrgan 2.0) **Amount of EVs in Bioink**: 4 × 10^9^ EV particles mL^-1^ **Bioink material**: GelMA **Bioprinting parameters**: printing speed = 200 mm min*-*1, *Q*bioink = 7 μL min*-*1, *Q*CaCl2 = 5.4 μL min*-*1). Ten-layer-thick, 200 μm hydrogel fiber at 10 *×* 4 *×* 1 mm^3^. **EV release experiment**: NTA for bioprinted EV media at 24 h, 48 h, 72 h, and 1 week	HUVEC-sEVs size: < 200 nm; more than 80% of bioprinted EVs remain in the constructs after 1 week ***In vitro***: Bioprinted HUVECs- EVs-SM hypoxia induced the formation of spindle-shaped multicellular structures in peripheral blood mononuclear cells (PBMCs) ***In vivo***: Subcutaneous transplantation of 3D bioprinted HUVECs-EVs led to new functional vasculature *in situ*, consisting of blood-perfused microvessels recapitulating the printed pattern
Yerneni *et al*.^[105]^	**Cell source**: murine J774A.1 monocytic cell line (M0 state) **CM condition**: maintained in Roswell Park Memorial Institute medium (RPMI, Gibco, Gaithersburg, MD) supplemented with 10% heat-inactivated fetal bovine serum (HI-FBS; Invitrogen, Carlsbad, CA), EV-depleted FBS obtained by centrifugation at 100,000 × *g* for 2 h and media was collected every 72 h. **sEVs isolation (Mini-SEC)**: 2,000 × *g* for 10 min at 4°C, 10,000 × *g* for 30 min at 4 °C. The supernatant was passed through a 0.22 µm-pore Millipore filter and EVs isolated by mini-SEC using 1.5 cm × 12 cm mini-columns (Bio-Rad, Hercules, CA, USA; Econo-Pac columns) packed with 10 ml of Sepharose 2B (Sigma-Aldrich, St. Louis, MO, USA) equilibrated with phosphate-buffered saline (PBS) **EV characterization**: Immunoblotting, TEM, SEC, TRPS, flow cytometry, Confocal microscopy	**Type of printing**: inkjet-based bioprinting technique **Amount of EVs in bioink**: 100 ug/mL eBMP2-EV **Bioink material**: consisting of 100 ug/mL eBMP2-EV in PBS and 10% glycerol **Bioprinting parameters**: coated coverslips (Neuovitro, Vancouver, WA) as 1.25 × 1.75 mm patterns arranged in 2 × 2 dose-modulated arrays of 5, 10, 15, and 20 OPs, with adjacent drop spacings of 80 µm. 1.75 mm × 1.25 mm **EV release experiment**: none	M0-sEVs size: ~100 nm BMP2 was effectively delivered into M0-EVs using sonification ***In vitro***: Bioprinted BMP-sEVs on collagen I-coated coverslips induced *in vitro* osteoblastogenesis in C2C12 cells compared to bioprinted EVs only ***In vivo***: Bioprinted BMP2-sEVs in the collagen-rich acellular dermal matrix (ADM) scaffolds induced localized heterotopic ossification in a mouse muscle pocket model
Sun *et al.*^[9]^	**Cell source:** Macrophages RAW 264.7 cell line stimulated by bioceramic (β-TCP) extracts **CM condition:** β-TCP extracts and 10% exosome-deprived FBS for 2 days and 4 days **EV isolation** (chemical-based precipitation using Invitrogen Total Exosome Isolation Reagent kit). 10,000 × *g* for 60 min **EV characterization:** TEM, NTA, BCA, and WB (CD9, CD81 TSG101)	**Type of bioprinting:** microextrusion **EV amount in bioprinting**: 100, 200, and 400 μg/mL bioceramic-induced macrophage-derived sEVs (BC-M0-sEVs) **Bioink materials:** 10% alginate and 5% hyaluronic acid (HA) **Bioprinting parameters:** not stated	BC-M0-sEVs size: ~110 nm ***In vitro*:** BC-M0-sEVs promoted the adhesion, migration, and immune response in macrophages. BC-M0-sEVs also enhance proliferation, survival, adhesion, osteogenic differentiation, and immunomodulation of hBMSCs and HUVECs
Yerneni *et al.*^[106]^	**Cell source**: a murine macrophage cell line (J774A.1) at M0 , M1 (LPS-treated) and M2 (IL-10 treated) status **CM condition**: Cells were cultured in M0, M1 and M2 RPMI medium containing 10% exosome depleted FBS (ED-HI-FBS) for 72 h **EV isolation (SEC)**: 2,500 × *g* for 10 min at 4 °C and 10,000 × *g* for 30 min at 4 °C, was followed by ultrafiltration (0.22 μm filter)and SEC using Bio-rad poly-Prep gravity-flow column **EV characterization**: BCA Protein assay, TEM, WB (CD63, CD9, and TSG101), DLS, and TRPS	**Type of printing**: inkjet-based system **Amount of EVs in Bioink**: 10, 50, 100, 200, and 300 μg/mL of sEVs in 0%, 1%, 5%, 10%, and 25% glycerol bioink **Bioink material**: glycerol. Final bioink formula 10 µg/mL sEVs with 10% glycerol **Bioprinting parameters**: speed of > 2 m/s without satellite drop formation; 10% glycerol (≥ 1 h print time). 25 µsec dwell at +12 V and 40 µsec echo at -12 V	Size of sEVs: ~ 100 nm. ***In vitro***: M0, M1 and M2-sEVs were bioprinted on collagen type-I coverslips. Bioprinted M1-sEVs solid microenvironments inhibited myogenesis. M2-sEVs solid-phase sEVs promoted myogenesis in C2C12 cells with upregulated skeletal muscle differentiation marker - myosin heavy chain II (MF20)
Yerneni *et al.*^[107]^	**Cell source**: THP1 and J774A.1 cell lines **CM condition**: both cells were cultured in HI-FBS, where HI-FBS was centrifuged at 100,000 g for 3 h, and CM was collected in EV-depleted supernatant was collected (ED-HI-FBS) for 48 h **EV isolation (SEC)**: 2,500 *g* for 10 min at 4 °C and 100,00 *g* for 30 min at 4 °C), followed by ultrafiltration (0.22 μm filter; and then size-exclusion chromatography on a Sepharose 2Bcolumn **EV concentration**: 20 µg (final concentration 0.2 μg/μL) **EV characterization**: dynamic light scattering, WB (TSG101, CD9, CD36), TEM, Flow cytometry; BCA, SEC, and tunable resistive pulse sensing (TRPS)	**Type of printing**: inkjet-printed **Amount of EVs in Bioink**: 100 μg/mL of Exo-ssDNA-SA-FasL sEVs in 10% glycerol for 50 overprints (OP) on collagen type-1 coated coverslips to create patterns of 1.25 mm × 1.75 mm corresponding to a total deposited sEVs protein concentration of 76 ng **Bioink material**: 10% glycerol **Bioprinting parameters**: 50 overprints were printed on the collagen type-1 coated coverslips with 1.25 mm × 1.75 mm to correlate with 76 ng sEVs protein; final density of 2.5 × 10^3^ cells/cm^2^ (overnight in PBS) **EV release experiment**: unclear	THP1 and J774A.1 -sEVs: 30-300 nm, mean diameter of 100 nm ***In vitro***: Bioprinted exo-ssDNA-SA-FasL triggered the apoptosis in cancer cell PCI-13 ***In vivo***: Intraperitoneal injections of Exo-ssDNA-SA-FasL inhibited the proliferation of donor CD4^+^ T cells in F1 mice
Chen *et al*.^[108]^	**Cell source**: Bone marrow-derived mesenchymal stem cells (BMSCs) **CM condition**: serum-free medium 300 × *g* for 15 min and 2,500 × *g* for 15 min **EV isolation (ultracentrifuge)**: 4,000 × *g* to concentrated to final volume 200 µL; 1h at 100,000 × *g* and 4,000 × *g* to 200 µL **EV characterization**: DLS, TEM, WB (TSG101, CD9, and CD63); internalization by chondrocytes	**Type of printing**: desktop-stereolithography (SLA) technology **Amount of EVs in Bioink**: 200 µg/mL **Bioink material**: GelMA, MSC-derived sEVs, and decellularized cartilage ECM **Bioprinting parameters**: 4 × 4 mm with different ECM ratios **EV release experiment**: SEM observation	MSC-sEVs size: 40-110 nm ***In vitro***: EMC/GelMA/MSC-sEVs scaffold promotes the regeneration of cartilage destruction ***In vivo***: a rabbit model was determined the ability and biosecurity of osteochondral defect models repair were enhanced *via* the scaffold Cylindrical defect 4 mm created in both limbs, 6 or 12 weeks after surgery analyzed by macroscopic and MIR

NTA: Nanoparticle tracking analysis; WB: Western Blot; FACS: flow cytometer; Enzyme-linked immunosorbent assay ELISA; TEM: Transmission electron microscopy; TSG101: Tumor Susceptibility Gene 101 Protein; HI-FBS: heat-inactivated fetal bovine serum; DLS: dynamic light scattering; BCA: Bicinchoninic acid; TRPS: tunable pulse resistive sensing; RPMI: Roswell Park Memorial Institute Medium; SEM: size-exclusion chromatography; THP1: human monocytic cell line derived from an acute monocytic leukemia patient; J774A. 1 cell: active in antibody-dependent phagocytosis.

### EVs characteristics before bioprinting

Conditional media collection and EV enrichment methods are key parameters of EVs characterization and downstream function. This review included 8 studies^[103-108]^ where EVs were enriched from cells cultured in either EV-depleted FBS supplemented^[105-107]^ or serum-free^[103,108]^ media. In terms of EV enrichment methods, SEC^[105-107]^ or ultracentrifuge^[103,108]^ was used for sEVs isolation. All isolated sEVs were smaller than 200 nm (i.e., confirmed as small EVs) after sEVs were characterized through common techniques - NTA, WB and TEM.

### Current studies of bioprinted sEVs as a regenerative medicine approach

**Figure 3:**
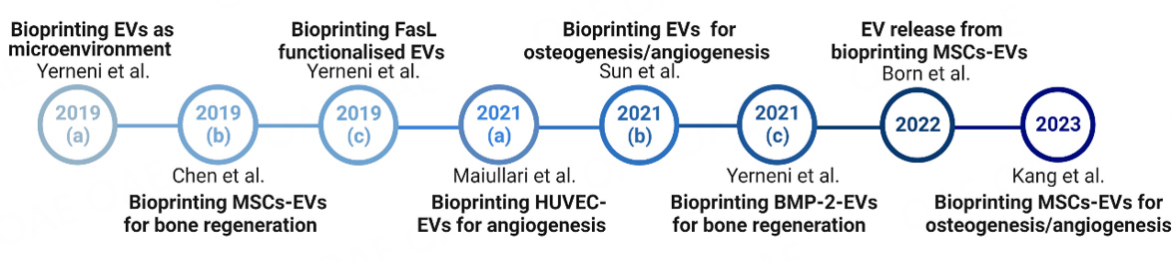
This review includes 8 research studies, as demonstrated by the timeline.

A recent review^[24]^ primarily focused on the future translational potential and applications of bioprinted sEVs. It proposed the concept of bioprinted multiphasic EV scaffolds for regenerative medicine. For instance, in the regeneration of osteochondral defects, the bioprinting of pro-osteogenic EVs, pro-chondrogenic EVs, and pro-angiogenic EVs into a scaffold can maintain the 3D environment^[24]^. While providing a summary of current applications, the review particularly highlighted the potential clinical implications and offered guidelines for future research studies.Thus, a detailed review of current studies is necessary to facilitate future researchers to fully understand the key parameters for a successful regenerative medicine outcome. This section detailed the current eight bioprinted sEVs studies^[9,103-109]^ [Figure 3], including cell source of sEVs, bioprinting technology, sEVs enrichment/characterization, application of bioprinted sEVs structures and key results of each study.

Theodoraki *et al.*^[102]^ explored the potential role of bioprinted EVs/ tumor-specific antibodies array to capture tumor-enriched EVs from HNSCC patients following oncological therapy. Tumor-derived (TEX)-EVs and T-cell-derived CD3+ EVs were isolated by SEC and immunocapture (CD3+ and CD- EVs) from plasma samples of 18 HNSCC patients after treatment with cetuximab, ipilimumab, and IMRT. After isolation, EVs were successfully characterized by TEM, BCA, and flow cytometry. The authors printed antibodies against CD9, CD63, CD81, EGFR1, MAGEA3, EpCAM and CSPG4 for sEVs capture in microarrays using an inkjet-based deposition system (glycerol as the viscosity modifier of the bioink). Five ug of total EV protein isolated from plasma samples was combined with printed antibodies and captured through microarray. The results showed that the levels of total EV protein and TEX-EVs, as well as CD3+, CD3(-)/PD-L1+ and CD3+15S+ EVs in patients with cancer recurrence, were significantly increased compared with baseline levels, while total EV protein and TEX levels decreased in patients without the disease. CD3+, CD3+/CD15S+ EVs were relatively stable, which further indicated that plasma EVs have certain advantages as tumor biomarkers in non-invasive tumor monitoring. This study supported the application of bioprinted EV antibody arrays as a powerful tool to isolate tumor enriched EVs subtypes. It is noted that this work is using bioprinted EVs-antibody array as a potential cancer diagnosis; it is not the therapeutic application of bioprinted EVs.

In one study, Kang *et al.*^[109]^ investigated the potential application of bioprinted human adipose-derived stem cell-derived extracellular vesicles (hADSCs-sEVs) for bone and angiogenesis *in vitro* and *in vivo*. The size of the sEVs was smaller than 200nm after NTA characterization, and they were printed into bioinks composed of decellularized extracellular matrix (dECM), gelatin (Gel), quaternized chitosan (QCS), and nano-hydroxyapatite (nHAp), forming what was referred to as dECM/Gel/QCS/nHAp@Exo scaffolds. Subsequently, hBMSCs were cultured on the bioprinted hADSCs-sEVs at different concentrations: 0, 10, 20, and 30 µg/mL. Notably, the 30 µg/mL group exhibited the highest levels of *in vitro* osteogenesis compared to the other groups. The release of extracellular vesicles over time after bioprinting showed that the peak EVs release occurred at 12 and 24 hours, and even after one week, approximately 16% of the EVs remained within the scaffolds, thereby promoting cell migration. To further evaluate the efficacy of bioprinted hADSCs-sEVs, an *in vivo* skull defect model was employed. After a ten-week period following surgery, the bioprinted hADSCs-sEVs at a concentration of 30 µg/mL were found to enhance bone formation and the development of blood vessels. Additionally, one week after subcutaneous implantation, these bioprinted sEVs demonstrated the ability to enhance angiogenesis. Collectively, these findings provide evidence that bioprinted hADSCs-sEVs have the capacity to promote osteogenesis and angiogenesis both *in vitro* and *in vivo*.

**Figure 4:**
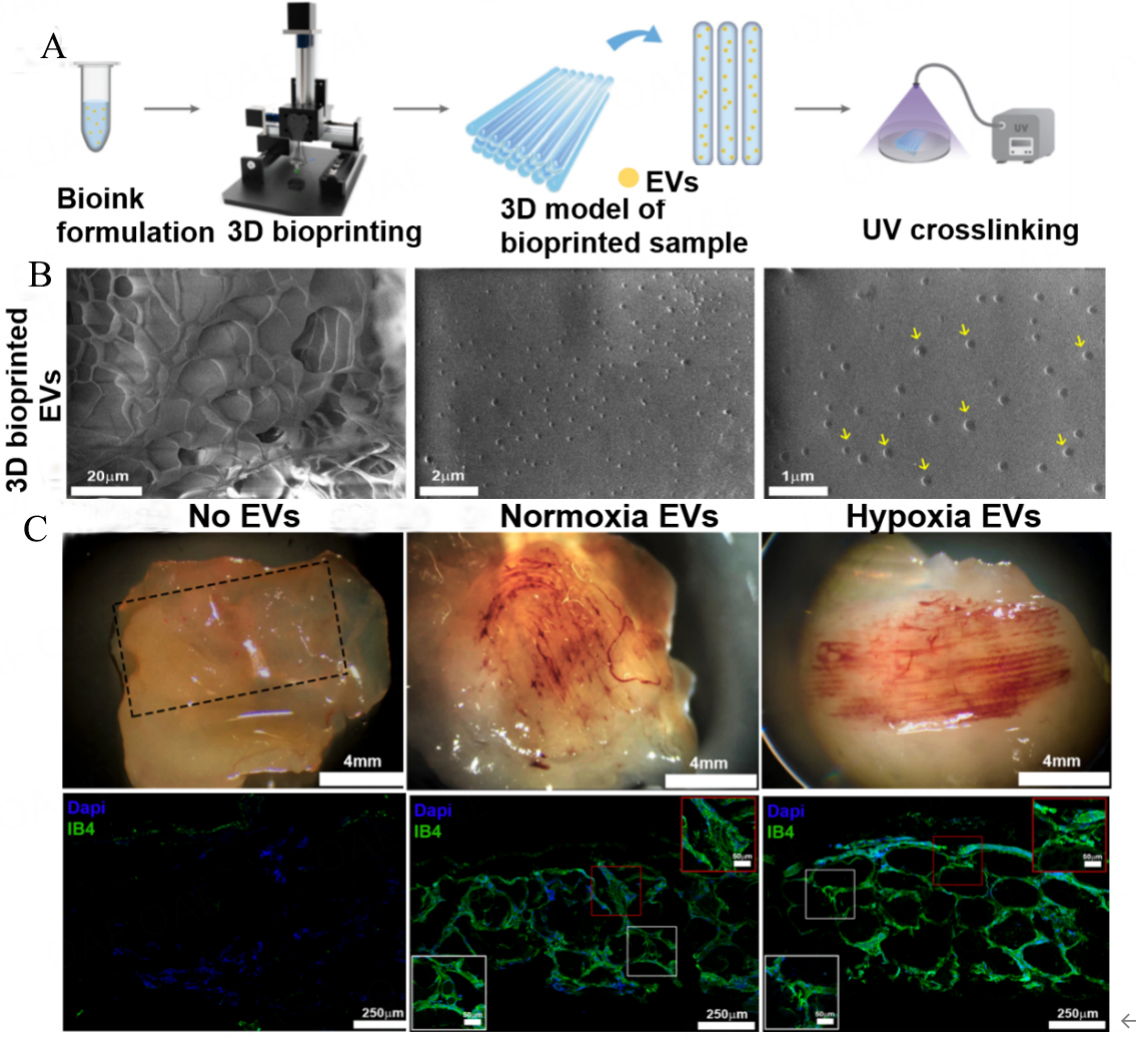
Bioprinted HUVECs-sEVs constructs promote angiogenesis (modified from ^[103]^). (A) Schematic representation of the bioprinting process to manufacture EV-loaded scaffolds; (B) SEM images of 3D bioprinted GelMA with HUVECs-sEVs. Yellow arrows denote the EVs; (C) Bioprinted HUVECs-sEVs promote *in vivo* vessel formation after subcutaneous implantation in immunocompromised mice, with IB4-positive blood vessel structures. IB4, Isolectin B4; Normoxia EVs, EVs from normoxia HUVECs; hypoxia EVs, EVs from hypoxia HUVECs.

A study by Maiullar *et al.*^[103]^ involved 3D bioprinted HUVEC-derived EVs for angiogenic applications *in vitro* and *in vivo* [Figure 4]. In this study, sEVs collected from culturing conditions of normoxia, hypoxia, serum-free normoxia and serum-free hypoxia (SM hypoxia) demonstrated an increased number of EV particles but reduced EV size under SM hypoxia conditions. The size of all sEVs was below 200 nm and bioprinted in gelatin methacrylate (GelMA) bioinks using the microextrusion bioprinter (CeciliaOpenOrgan 2.0) technique. Nanoparticle tracking analysis (NTA), Western blot (CD9, CD81), FACS (CD9, CD81, and CD63), ELISA (VEGF, PIGF, VEGFR1, VEGFR2) and TEM analysis were performed to characterize the HUVEC-derived EVs. According to *in vitro* culture of the bioprinted structures under SM Hypoxia in EV-free media for 7 days, more than 80% remained in the constructs after 1 week. The results showed that bioprinted HUVECs-SM hypoxia-EVs enhanced the production of spindle-shaped multicellular structures in peripheral blood mononuclear cells (PBMCs) *in vitro*. In an *in vivo* model, bioprinted EVs were subcutaneously implanted in NSG and C57/BL6 mice (*n* = 3) and neovascularization was evaluated after 60 days. The bioprinted SM Normoxia-EVs and SM Hypoxia-EVs groups resulted in functioning neovasculature with secondary branches that cross-connect the larger veins. These results showed that bioprinted HUVEC-EVs facilitated the incorporation of functional vasculature in situ, including blood-perfused microvessels that matched the printed pattern.

Born *et al.*^[104]^ optimized the efficacy of printing MSCs-EVs in GelMA bioink with different concentrations of LAP. MSCs-derived EVs were isolated by ultracentrifugation and characterized by TEM and Western blot (CD63, TSG101, and Alix). The size of MSC-EVs ranged from 30 to 250 nm (mean = 130 ± 51 nm). Bioprinted EV-GelMA crosslinked with 0.1% LAP displayed a significant EV release, while the release from 0.2% LAP crosslinked gels was prolonged over the first 3 days, but completed by 14 days for both groups. An in vitro endothelial gap closure assay showed that the bioprinted EVs constructs significantly increased HUVEC response at 1-day post-incubation. The results demonstrated that bioprinted MSC-EVs can promote endothelial cell migration *in vitro*.

**Figure 5:**
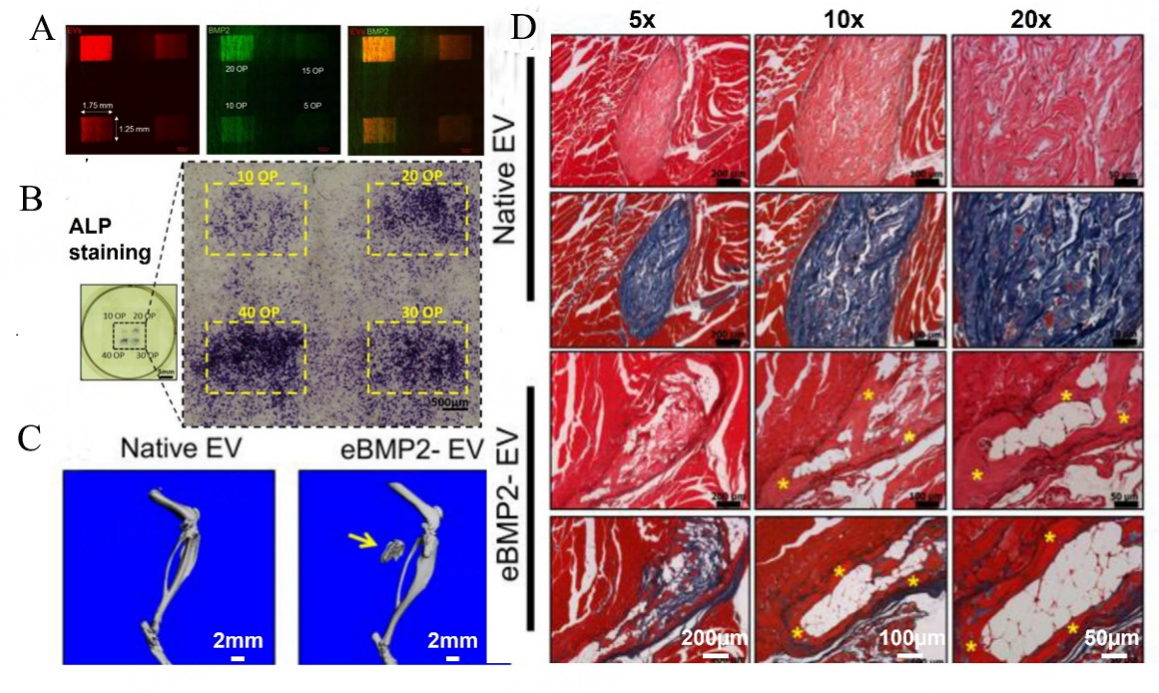
*In vitro* and *in vivo* assessment of bioprinted BMP2-EVs (modified from^[105]^). (A) Bioprinted patterns of Alexa Fluor 488-labeled BMP2 (green) loaded in PKH26-labeled EVs (red); (B) ALP staining of C2C12 cells at 72 h post-seeding on bioprinted BMP2-EVs patterns with indicated OPs; (C) Representative microCT 3D reconstructions of mouse leg scans containing either native EVs or BMP2-EVs bioprinted implants. Arrow points to heterotopic ossification; (D) Representative histological images showing H&E and Masson's trichrome staining of native EVs and BMP2-EVs bioprinted implants (*indicates bone tissue).

Yerneni *et al.*^[105]^ reported *in vitro* and *in vivo* osteogenic bioactivity with bioprinted eBMP2-EVs [Figure 5]. EVs were isolated from the murine J774A.1 monocytic cell line (M0 state) by the SEC method, and M0-EVs size was approximately 100 nm. Electroporation or sonication was used to load bone morphogenetic protein-2 (BMP2) into the lumen of M0-EVs (eBMP2-EV) (10 μg EVs and 1 μg 125I-BMP2 protein), where sonication led to 3-fold higher loading efficiency than electroporation. Bioprinted eBMP2-EV solid-phase microenvironments were then created on collagen-coated coverslips using an inkjet-based bioprinting technique consisting of 100 ug/mL eBMP2-EV in PBS and 10% glycerol bioink. The *in vitro* assay for these bioprinted BMP2-EV solid microenvironments demonstrated enhanced osteogenic ALP activity (key osteogenic marker) in C2C12 cells after 3 days. In an in vivo study, the investigators bioprinted and implanted eBMP2-EVs or EVs in collagen-rich acellular dermal matrix (ADM) scaffolds (5 ng BMP2 + 150 ng EVs per 4.5 mm ADM disc) into thigh muscle pockets of male C57BL/6 mice. eBMP2-EV ADM constructs induced a greater degree of heterotopic ossification compared to EVs only at 4 weeks post-implantation. This study demonstrated that bioprinted BMP-M0-EVs can enhance osteogenic mineralization *in vitro* and *in vivo*.

A study by Sun *et al.*^[9]^ investigated the effects of bioceramic-induced macrophage-derived sEVs on the cellular response of M0 macrophage, hBMSCs and HUVECs. The sEVs were isolated from the RAW 264.7 macrophage cell line through a chemical-based precipitation method called Total Exosome Isolation Reagent. These sEVs were obtained by stimulating the RAW macrophages with extracts of a specific bioceramic known as β-tricalcium phosphate (β-TCP), resulting in what was referred to as BC-M0-sEVs. The BC-M0-sEVs were approximately 110nm in size and then were bioprinted onto a scaffold composed of 10% alginate and 5% hyaluronic acid (HA) at three different concentrations: 100, 200, and 400 μg/mL. The study findings demonstrated that the bioprinted BC-M0-sEVs positively influenced migration, attachment, and immune response in M0 macrophages, hBMSCs, and HUVECs *in vitro*. Additionally, the bioprinted BC-M0-sEVs scaffolds exhibited enhanced osteogenesis in hBMSCs and angiogenesis in HUVECs. These results indicate the potential of bioprinting BC-M0-sEVs for applications involving tissue engineering and regenerative medicine.

Another research used bioprinted murine macrophage-derived EVs as an *in vitro* extracellular matrix (ECM) microenvironment^[106]^. EVs were enriched using an SEC column from a murine macrophage cell line (J774A.1) at M0 (non-activated, M0-EVs), M1 (pro-inflammatory, M1-EVs), and M2 (pro-regenerative, M2-EVs) phenotypes. All EVs were approximately 100 nm in size and bioprinted in glycerol bioink on collagen type-I-coated glass slides using a custom inkjet-bioprinter. The bioink overprints (OPs) with different EV amounts (0.1, 0.2, 0.95, and 12 µg EV protein) were defined as 10, 20, and 40 OPs, respectively. Myogenesis in murine myoblast C2C12 cells was used to assess the impact of bioprinted microenvironments on cell bioactivity. The results showed that the bioprinted M1-EVs microenvironment inhibited, while M2-EVs promoted, myogenesis in C2C12 cells via upregulation of myosin heavy chain (MF20) expression and the formation of myotubes. Together, these findings demonstrated the promotion of myogenesis by bioprinted M1-EVs *in vitro*.

Again, Yerneni *et al.*^[107]^ bioprinted oligonucleotide-tethered macrophage-derived sEVs (Exo-ssDNA-SA-FasL) for tumor cells apoptosis *in vitro* and immunomodulation *in vivo* that promoted an anti-cancer function. THP1-sEVs were isolated using the SEC method, with a size of approximately 100 nm. Single-stranded DNA (ssDNA) with a conjugated cholesterol moiety was tethered to the sEVs lipid bilayer via Fas-ligand fusion with a modified version of streptavidin (SA-FasL), where FasL is an immunomodulatory protein binding with Fas receptor that induces apoptosis. Exo-ssDNA-SA-FasL (100 μg/mL) was bioprinted in 10% glycerol bioink using inkjet-based bioprinting. Bioprinted Exo-ssDNA-SA-FasL solid microenvironment triggered FasL/Fas-mediated apoptosis in the squamous cell carcinoma of the head and neck (SCCHN) cell line PCI-13 *in vitro*, with a significant number of dead cells compared to bioprinted sEVs only. In an in vivo model, 40 μg of Exo-ssDNA-SA-FasL was delivered by intraperitoneal (i.p.) injection to C57BL/6-DTR / BALB/c-crossed mice. At 72 h post-injection, the Exo-ssDNA-SA-FasL group expressed significantly fewer numbers of CD3+ and CD4+ T cells in the spleen, and CD4+ T cells in lymph nodes, compared to sEVs controls. This study demonstrated that bioprinted Exo-ssDNA-SA-FasL increases cancer cell apoptosis in vitro and eliminates alloreactive T cells *in vivo*.

**Figure 6:**
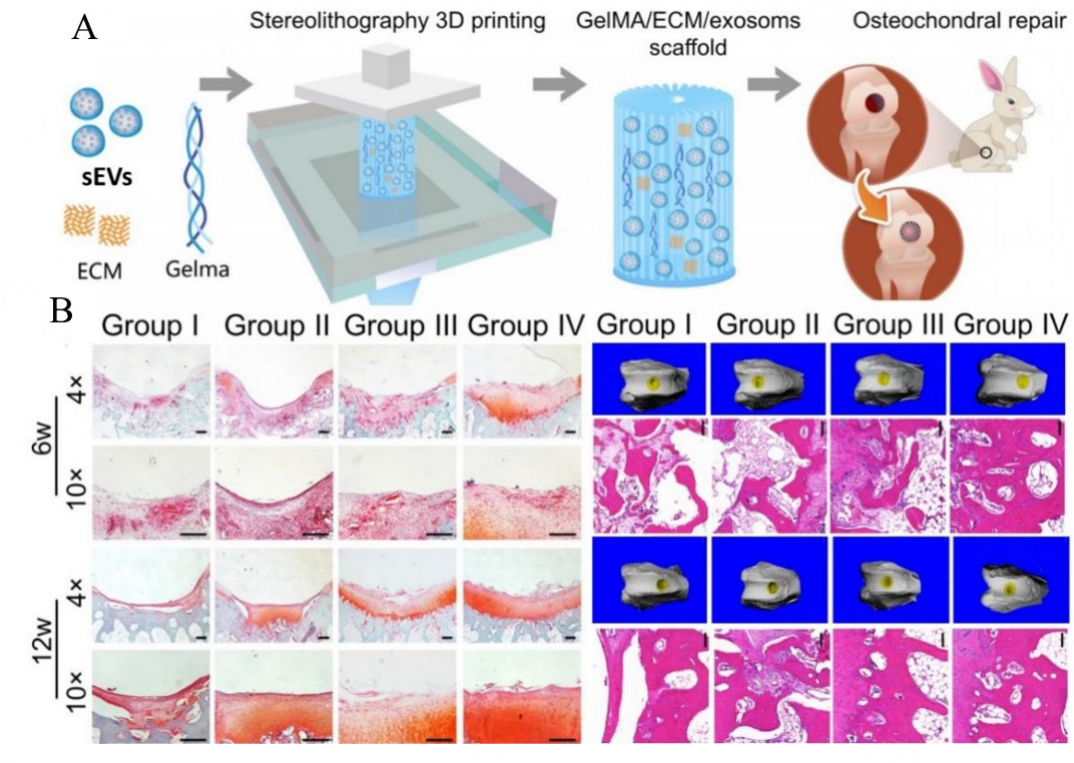
Bioprinted MSCs-sEVs constructs facilitate both cartilage and bone regeneration in a rabbit (modified from ^[108]^). (A) Schematic illustration of stereolithography-based bioprinted MSCs-sEVs in decellularized cartilage ECM and GelMA bioink prior to *in vivo* osteochondral defect implantation in a rabbit. SEV is known as sEVs; (B) Bioprinted MSCs-sEVs promoted both cartilage and bone formation after 6 and 12 weeks of implantation. Group I, osteochondral defect only; Group II, 3D bioprinted GelMA; Group III, 3D bioprinted ECM/GelMA scaffold; Group IV, 3D printed ECM/GelMA/sEVs scaffold.

Another study printed MSCs-sEVs in cartilage ECM and GelMAbioink that induced cartilage and bone regeneration *in vivo*^[108]^ [Figure 6]. sEVs generated from bone marrow-derived mesenchymal stem cells (BMSCs) were bioprinted and applied to an osteochondral defect. The MSCs-EV size ranged from 40-110 nm and bioprinted in photo-crosslinked decellularized pig cartilage ECM and GelMA bioink by desktop-stereolithography technology. The bioprinted scaffold retained 56% of sEVs for 14 days in vitro, and for 7 days *in vivo* following subcutaneous implantation in a rat model. *In vitro*, bioprinted ECM/GelMA/MSC-EVs scaffolds promoted chondrocyte migration, while *in vivo*, significantly increased and decreased numbers of ARG-I+ and CD163+ M2 macrophages, and CD86+ M1 macrophages, respectively, were present in surrounding tissue 1-week post subcutaneous placement of ECM/GelMA/sEVs scaffolds. In the same study, implantation of 3D printed ECM/GelMA/sEV scaffolds into a rabbit osteochondral defects model resulted in enhanced neo-cartilage-like tissue and subchondral bone formation at 6 and 12 weeks, determined by MIR screening and HE staining, compared to the ECM/GelMA control group. It was found that radially oriented ECM/GelMA/sEV scaffolds successfully restored cartilage mitochondrial dysfunction, enhanced chondrocyte migration, and polarized the synovial macrophage response towards an M2 phenotype. This work showed that bioprinted MSC-EVs in cartilage ECM and GelMA enhanced both cartilage and bone formation *in vivo*.

It is important to highlight that among the analyzed eight studies, diverse amounts of sEVs were employed for the bioprinting, ranging from 8.84 µg/mL^[104]^, 30 µg/mL^[109]^**,** 100 μg/mL^[105,107]^, 200 µg/mL^[106,108]^ to 400 µg/mL^[9]^, with one study utilizing 4 × 10^9^ EV particles per mL^[103]^**.** The wide range of EV amounts employed in bioprinting studies may play a significant role in the observed variations in EV release profiles among different investigations. The varying concentrations of EVs utilized during the bioprinting process might directly impact the kinetics and extent of EV release, subsequently influencing the overall release profiles reported in each study. The differences in EV concentrations present a plausible explanation for the observed variability in EV release profiles across the examined studies.

### Summary and discussion

It is noted that among the eight studies, five studies applied bioprinted sEVs in pre-clinical animal work^[103,105,107-109]^. More studies are needed to further validate the in vivo functional role of bioprinted sEVs. This review focuses on current studies supporting 3D bioprinted sEVs in bioinks such as GelMA and glycerol for various tissue engineering approaches [Figure 6]. Both *in vitro* and *in vivo* models demonstrate that bioprinted MSCs-sEVs can promote cell migration, cartilage regeneration, and angiogenesis. 3D bioprinting of either macrophage (M0 or M2)- or monocyte (THP1)-derived sEVs can facilitate bone regeneration, myogenesis, and immunomodulation. These outcomes support the notion that sEVs retain the same functional roles as their parent cells. However, more studies of other cell-derived sEVs are required to confirm that these observations can be applied to a wider range of therapeutic applications.

Although bioprinted sEVs hold great promise for tissue engineering applications, several challenges need to be taken into account:

(1) Technical challenges remain a significant issue - from sEVs isolation and characterization to standardization of clinically suitable sEVs preparations (reviewed i^[28,33]^). Among the seven studies included in this review, four studies used SEC and three utilized UC, while UC is a low throughput method that may yield contaminated sEVs populations. Only two studies used primary cell - MSC, and most of the studies employed cell lines; this may be an issue for future clinical translation as cell lines are not always good representatives for a real clinical scenario. The low yield of cell-derived sEVs is another challenge in the field; thus, scalable cell cultures, such as using a bioreactor or 3D scaffolds, can be an alternative way to achieve cell culture scalability. More importantly, the MISEV guidelines stipulate that EV researchers should specify EV purity (i.e., EV particle numbers per ug protein) and enzyme treatment (DNase, RNase and proteinase-treated sEVs to increase the EV purity) information as essential considerations for clinical translation.

(2) sEVs can be employed as drug delivery agents; one study^[105]^ showed that loaded BMP2 into M0-sEVs and then bioprinted M0-BMP2-sEVs were able to increase in vivo bone formation. However, a question that arises is whether loading exogenous cargo interacts with endogenous cargo and whether this creates issues associated with off-target effects.

(3) Whether bioprinted sEVs can perform their therapeutic application at targeted sEVs delivery sites with an appropriate release profile is another important aspect to investigate. Sustained sEVs release is a key factor to consider when designing bioinks and their mechanical properties for released sEVs targeting specific cells. Additionally, the selected bioink materials and bioprinted structures should be ideally degraded after sEVs are released to desired sites for host cell recruitment.

(4) Functionalizing EVs and their binding to the printed scaffolds are vital considerations for achieving targeted cellular responses after the sustainable release of EVs from bioprinted scaffolds. In a study^[107]^, EVs were functionalized with FasL before being bioprinted into a scaffold, resulting in targeted binding to recipient cancer cells and eliciting specific responses. The specific binding of EVs to the printed matrix is crucial for well-defined retention fidelity of printed patterns, surpassing diffusion-based approaches. Incorporating surface modifications and specific binding mechanisms allows precise immobilization of EVs within the matrix, ensuring spatial arrangement and functional distribution control. This advancement holds great promise for enhancing bioprinting applications in tissue engineering and regenerative medicine. All the above-mentioned points are critical to achieving optimal tissue regenerative outcomes and future bioprinted EVs studies should consider these factors. Although there are four pre-clinical studies, it is still at its early stage to validate the potential of bioprinted EVs in regenerative medicine since more future studies are required to include more primarily cells sourced EVs and various tissue regeneration applications.

## CONCLUSION

**Figure 7:**
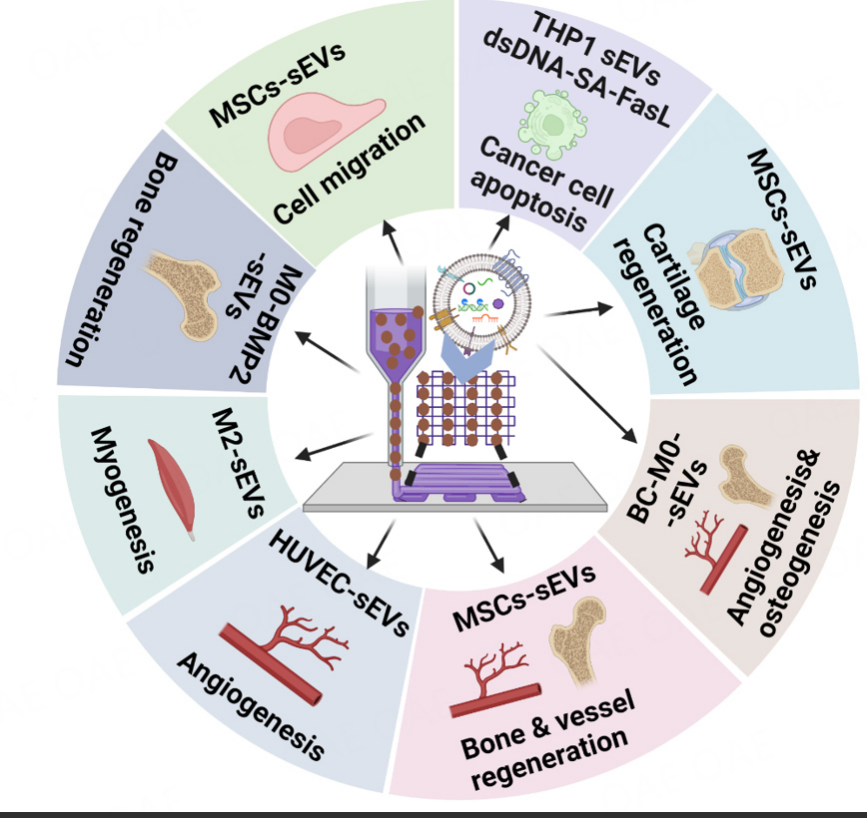
Summary of sEVs from different cell sources and functions of bioprinted sEVs as "cell-free" regenerative medicine approaches. MSC-sEVs: MSC-derived small EVs; HUVEC-sEVs: Human umbilical vein endothelial cell-derived small EVs; M0-BMP2-sEVs: inactivated macrophage (M0) derived sEVs loaded with BMP2 protein; M2-sEVs: Macrophage stage 2 small EV (pro-regenerative); THP1-sEVs-dsDNA-SA-FasL: human monocyte cell line THP1 derived sEVs tettered with dsDNA and modified with streptavidin (SA) and Fas Ligand (FasL); BC-M0-sEVs: bioceramic-induced macrophage-derived sEVs.

Despite ongoing unresolved challenges, significant advances in bioprinting of sEVs have occurred in recent years. Indeed, as reviewed here, a small number of pioneering bioprinted sEVs strategies have been shown to be effective as "cell-free" regenerative medicine means [Table 2 and Figure 7]. Increasing interest has set the stage for the potential translational application of bioprinted sEVs as novel regenerative medicine approaches.

Inspired by the current personalized medicine concept^[24,110]^, personalized bioprinted EVs may be the future of clinical translational applications^[111]^. Here, we propose that patient-specific defects can be scanned *via* CT or X-ray technologies. Then, personalized computer-aided design (CAD) modeling can be employed to fabricate personalized bioprinted EVs constructs to mimic defect-specific. The last step is to apply the personalized EVs scaffolds to the patient. However, EVs enrichment, characterization and bioprinting optimization remain a significant technical challenge; the personalized bioprinted EVs concept may take some time to fully develop towards clinical translation. Thus, further development and studies require technical advances, such as scaling up in production, suitable bioink choice and targeted/controlled delivery, that are required for future clinical use. Although these technical hurdles remain to be addressed, the recent rapid growth in bioprinted sEVs has revealed many potential therapeutic applications, paving a path towards the realization of their vast clinical potential.

## DECLARATIONS

### Availability of data and materials

Raw data is available from the corresponding author upon reasonable request.

### Copyright

@ The Author(s) 2023.
